# Animal trypanosomosis in clinically healthy cattle of north Cameroon: epidemiological implications

**DOI:** 10.1186/s13071-016-1498-1

**Published:** 2016-04-13

**Authors:** Abdoulmoumini Mamoudou, Alexandre Njanloga, Aliyou Hayatou, Pierre Fongho Suh, Mbunkah Daniel Achukwi

**Affiliations:** Department of Parasitology and Parasitological Disease, School of Veterinary Medicine and Sciences, University of Ngaoundéré, P.O. Box 454, Ngaoundéré, Cameroon; Department of Animal Biology and Physiology, Parasitology and Ecology Laboratory, Faculty of Science, University of Yaounde I, P.O. Box 812, Yaounde, Cameroon; Institute of Agricultural Research for Development (IRAD), Wakwa Regional Centre, Ngaoundéré, P.O. Box 65, Ngaoundéré, Cameroon; TOZA Research Foundation, P.O. 59, Bambili, North West Region Cameroon

**Keywords:** Animal trypanosomosis, Clinically healthy cattle, Prevalence, Tsetse flies, North Cameroon

## Abstract

**Background:**

The control of animal trypanosomosis consists, amongst other things, of the punctual treatment of new cases, primarily diagnosed by pastoralists on the basis of clinical signs. This practice suggests that many apparently healthy infected animals are left untreated. In this study animal trypanosomosis in clinically healthy zebu cattle was evaluated, the distribution of the vectors established and the epidemiological implications discussed.

**Methods:**

In 2014 two cross-sectional surveys were carried out in the Cambeef ranch. A total of 866 blood samples were collected from cattle in different sites: 549 in the dry season and 317 in the rainy season. The blood samples were subjected to parasitological examination using the buffy coat method and to PCV determination. An entomological survey on animal trypanosomosis vectors was undertaken during tsetse flies caught were identified and the mid-gut of each living non-teneral tsetse fly was examined for infections using a microscope.

**Results:**

An overall trypanosomosis prevalence of 9 % was found in the cattle examined. There were significantly (*P* < 0.05) more trypanosome infected cattle in the dry season than the rainy season. Trypanosome-infected cattle had significantly (*P* < 0.05) lower Body Condition Scores (BCS) and Packed Cell Volumes (PCV) in the dry season than in the rainy season. Anemia was positively correlated with trypanosome infection. The likelihood for an animal to be parasitologically free of trypanosome infection was at least three times as high in the Gudali breed as compared with the white and red Fulani breeds. Species of trypanosomes identified were *Trypanosoma vivax* (73.23 %), *Trypanosoma congolense* (15.49 %) and *Trypanosoma brucei* (11.27 %). A total of 390 tsetse flies and 103 tabanids were trapped. Two species of tsetse flies were identified: *Glossina tachinoides* (33.59 %) and *G. morsitans submorsitans* (41 %). Nine of the 194 non-teneral flies were infected with trypanosomes.

**Conclusion:**

Carriers of trypanosomes are present amongst apparently healthy cattle in the study site. Attempts to successfully reduce the population of reservoir trypanosomes within herds and control the disease will need to consider mass screening once every year and this should be associated with drug sensitivity tests.

## Background

The Mayo Rey division is the largest pastoral zone of the north region of Cameroon [1]. Livestock production in this zone is, like in many other areas in Africa, impeded by the presence of tsetse fly [2] which cyclically transmits animal trypanosomosis [3]. This disease contributes to the impoverishment of pastoralists by causing abortion, premature births, prenatal losses, infertility in males through testicular damage, the reduction of milk production in diary animals and increased spending on drugs (trypanocides). According to the FAO [4] about 60 million cattle and 100 million small ruminants are exposed to the risk of the disease. Direct losses and cost of animal trypanosomosis control is estimated to range between 600 and 1,200 million USD per year for sub-Saharan Africa [5]. This disease is alone responsible for one quarter of economic losses due to animal pathologies [6]. The use of trypanocides has remained the main control measure for most African pastoralists. The risk of trypanosomosis transmission for most countries occurs between the end of the rainy season and the beginning of the cold dry season. It is recommended that all cattle be administered trypanocides, two weeks before these periods. Highly susceptible cattle receiving Isometamidium and trypanotolerant cattle diaminazene aceturate (3.5 mg/kg). Thus all animals should be protected during this period. Beyond these periods, sporadic cases, generally diagnosed by herdsmen on the basis of clinical signs solely, are treated with diaminazene aceturate at 7 mg/kg [7].This practice suggests that many apparently healthy but infected animals are left untreated and may contribute to the persistence of the disease. In this study we evaluate the prevalence of animal trypanosomosis in zebu cattle apparently showing no sign of illness; compare health indicators, describe the trypanosomosis vector distribution in Cambeef ranch and discuss implications for the epidemiology of the disease.

## Methods

### Study site

**Fig. 1:**
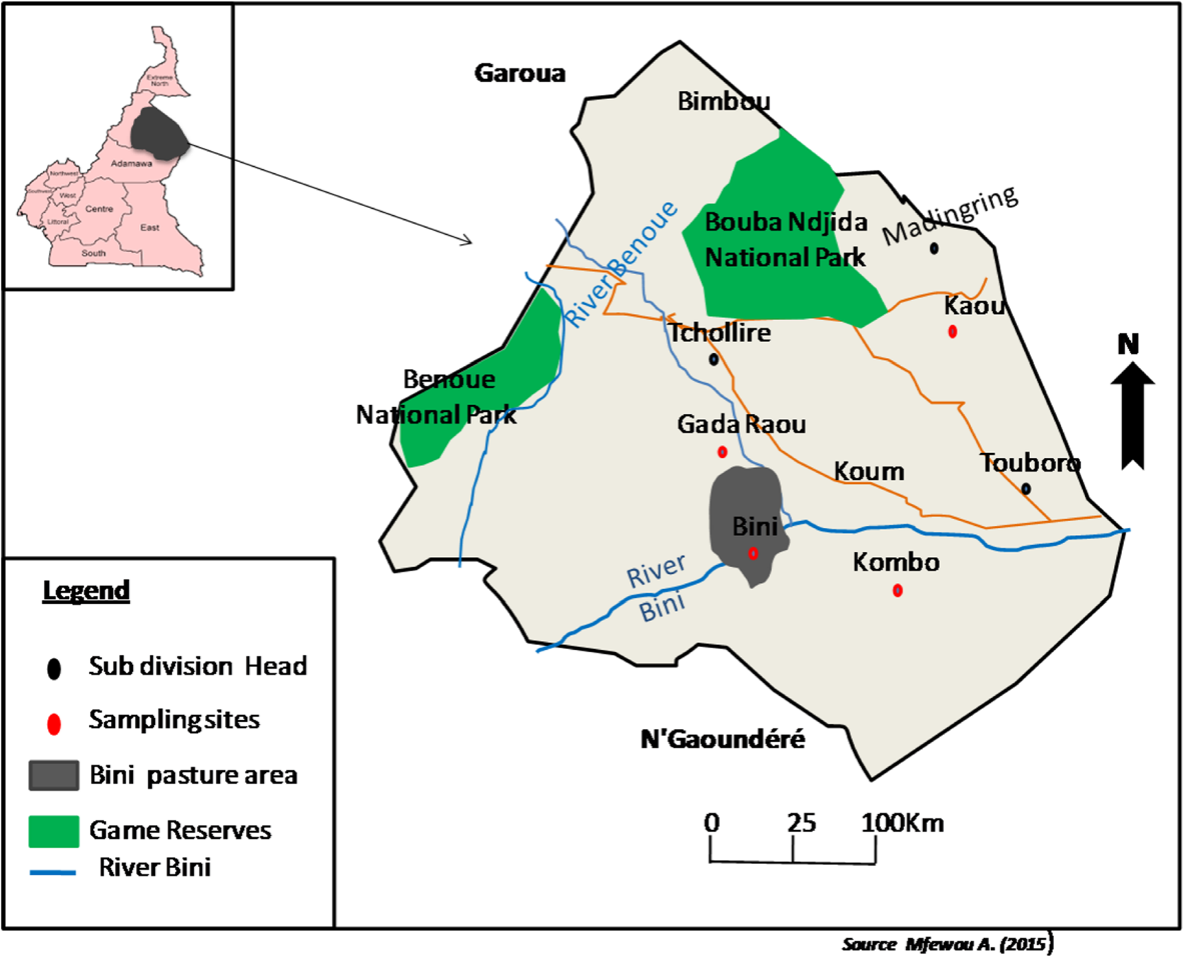
Map of study Area showing sampling sites

The Mayo-Rey is in the Sudano-sahelian zone covering about 36.524 km^2^ with a human population estimated at 11,454 inhabitants. It is located in the north region of Cameroon between Latitude 8°47′00″ N and 14° 01′00″ E (Fig. 1). There are two main seasons: the rainy season (early May to September) and the dry season (October to April); the main annual rainfall varies between 1,000–1,500 mm [8]. The vegetation is dominated by herbaceous plants [8]. The hydrographic network is made of the Benue and the Logone rivers and their tributaries. The flow of these rivers depends amongst other things on the abundance of rains and factors related to the soil [9]. The Mayo Rey is a large agro-pastoral zone. Its pasture is abundant and composed of *Andropogon gayanus, Brachiaria bryzantha, Loudetia togoensis* and *Pennisetum pedicellatum* which attract many transhumant pastoralists. Areal pasture composed of ligneous plants offers livestock feed resources during the dry season. Pastoralists are mainly of the Peuhl ethnic group [10]. The livestock farming system is mostly traditional and a few pastoralists practice the ranching system, amongst them is the Cambeef ranch. This ranch is about 100 km^2^ and divided into four sectors Kaou (15herds), Gada Raou (22 herds), Kombo (9 herds) and Bini (21 herds) (Fig. 1). It has stallings, water points, animal restraint corridors etc. Herdsmen take the 67 herds of the ranch to the pasture every day. Cattle were vaccinated against major epizootics (contagious bovine pleuropneumonia, black quarter, Lumpy skin disease and pasteurellosis). They were regularly administered anti-helminthics and every three months they received a preventive treatment of Isometamedium against trypanosomosis. Animals suspected of trypanosomosis were usually treated with diaminazene. A team of veterinary technicians and a veterinary doctor regularly checked the health of the herds.

### Study design

Blood was collected from cattle grazed in all the four sectors of the Cambeef ranch.

Of the 67 herds of the ranch, 29 were selected randomly in the dry season and 24 were selected in the rainy season. The number of herds selected with respect to the zones was as follows: 6, 8, 2 and 13 from Kaou, Gada Raou, Kombo and Bini, respectively, in the dry season and 4, 8, 3 and 8 in the same order in the rainy season. The average size of a herd was 70. Animals included in the survey were those that had no clinical sign of infection at least two months after receiving treatment with trypanocides. The eye color chart was used to exclude cattle with anaemia. Herdsmen were involved in the selection process. The weight of each animal was estimated by the formula developed by Njoya et al. [11]; the Body Condition Score (BCS) was determined according to Enzanno et al. [12] and categorized as follows: BCS Category: Poor:≤ 3; Good: > 3 and < 7; and Very good: ≥ 7. The survey was undertaken between November and January 2013 and repeated between May and June 2014.

### Bovine trypanosomosis survey

The blood was collected from the jugular vein of animals into Ethylene Diamine Tetraacetic acid (EDTA) tubes. While in the field, samples were kept in a container with ice packs and transported to the laboratory. In the laboratory, capillary tubes were filled with blood and one end of each capillary tube was sealed with cristaseal before centrifugation. The Packed Cell Volume (PCV) was subsequently measured and recorded [13]. Animals with PCV value below 24 % were considered to be anaemic [14]. The Buffy coat was extruded on microscope slides, covered with a coverslip and examined with a dark-field microscope to find trypanosomes. Parasitaemia was estimated according to the method defined by Murray et al. [15] and trypanosome species were identified by reference to the criteria defined by the same authors.

### Entomological survey

The entomological survey was undertaken in the dry season from November 2013 to March 2014. Two types of traps were used: the Nzi traps (*n* = 5) and biconical traps (*n* = 10). These traps were pitched around water points and forest galleries and in areas which herdsmen reported to be highly infested by tsetse. The geographical position of each trap was recorded using a Global Positioning System [GPS eTrex®; Garmin (Europe) Ltd, Southampton, UK]. Traps were exposed for three consecutive days and visited twice daily so as to avoid trapped flies from drying up. Acetone was used to increase the attractiveness of traps. At fly harvesting, fly catch cages were wrapped in humid tissue and placed in a cooled container in order to keep the flies alive and were then transported to the laboratory for identification. Tsetse flies and other haematophagous flies were identified by morphological criteria [16–21]. They were counted and sorted into teneral and non-teneral tsetse.

### Determination of trypanosome infection in tsetse flies

Each of the living non-teneral tsetse flies was placed on a glass slide and dissected in a drop of 0.9 % saline solution using a stereo-microscope. The wings were removed; the fly was immobilized with tongs; the proboscis was thereafter removed, followed by the salivary glands and the midgut. They were examined under a light microscope at a magnification of × 100 for the presence of trypanosomes. After processing each tsetse fly, the dissecting instruments were carefully cleaned by immersing them in a solution of 0.1 M sodium hydroxide and then, in distilled water.

### Data analyses

Chi-square and Z tests were used to compare the prevalence of trypanosome infections in the animals and trypanosomes species. The mean PCV and BCS of trypanosome infected and uninfected cattle were compared by Student’s *t*-test. Odds ratios (OR) were used to assess the association of trypanosomosis and PCV. The Apparent density of fly per trap and per day (ADT) was calculated using the following formula:

$$ \mathrm{A}\mathrm{D}\mathrm{T}\kern0.5em =\kern0.5em \frac{N}{TxD} $$

where N is the total number of flies caught, T is the number of traps deployed and D is the duration of trapping in days.

The ADTs were computed according to zones, type of trap and altitude (two modalities: ≤ 730 m or > 730 m). The Student’s t-test and F-test were used to compare means. The software SPSS version 17.0 was used to perform all statistical tests. Level of precision was held at 95 % and *P* ≤ 0.05 set for significance.

### Ethical approval

Necessary permissions from the Department of Parasitology and Parasitological Diseases of the School of Veterinary Medicine and Sciences of the University of Ngaoundere, Cameroon were taken to conduct the research. A verbal consent was obtained from the herders and cattle owners, with care taken in blood collection in order not to harm the animals. Motivations were made, which also included treatment of sick animals after the study.

## Results

A total of 866 animals were examined: 549 in the dry season and 317 in the rainy season; these comprised more female (564; 65.12 %) than male (302; 34.87 %). The BCS of animals was globally good and stood at 3.51 (standard deviation, SD = 0.36); the mean BCS was significantly (*t*_(44)_ = −6.51, *df =1, P* = 0.0001) higher in the rainy season (3.42; SD = 0.56) than in the dry season (3.18; SD = 0.50). The mean PCV was 33.59 (SD = 4.55). The mean PCV was significantly (*t*_(44)_ = 2.19; *df =* 4*, P* = 0.029) higher in the rainy season 33.92 (SD = 6.03) than the dry season 33.03 (SD = 4.69). Thirty nine cattle were anaemic (PCV ≤24 %). There were more anaemic cattle in the dry season (5.28 %; 29/549) than the rainy season (3.15 %; 10/317), but the difference was not significant (*χ*^*2*^ *=* 2.116, *df =* 4*, P* = 0.146).

### Prevalence of trypanosome infections in cattle

Of the 866 animals examined, 78 (9.00 %) were infected with trypanosomes: 67 during the dry season and 11 during the rainy season, corresponding to a prevalence of 12.20 and 3.47 % for the dry and rainy seasons, respectively. These frequencies were statistically different (*Z =* 4.35, *P* = 0.0001). Parasitaemia varied between 10^2^ and 10^6^ trypanosomes per ml of blood. The majority of infected cattle (71.43 %) had a parasitaemia varying between 10^4^ and 5 × 10^5^ trypanosomes per ml.

The highest trypanosomosis prevalence was recorded in Bini (11.27 %; 39/346), followed by Kombo (10.14 %; 7/69), Gada Raou (9.71 %; 17/175) and Kaou (8.52 %; 15/176) but these proportions were not significantly different (*χ2 =* 1.02, *df =* 4*, P* = 0.796). The prevalence was significantly higher in the dry season than in the rainy season for all the zones except Kombo (Table 1).

**Table 1:** Prevalence of trypanosome-infected cattle per zone and season in the Cambeef ranch

Zones	No. examined	No. positive (%)	*Z*, *P*-value
Bini	240	34 (14.16) (d)	2.538, 0.011
	106	5; 4.72 (r)	
Kombo	39	3; 7.69 (d)	−0.769, 0.441
	30	4; 13.33 (r)	
GadaRaou	155	16; 10.32 (d)	3.240, 0.001
	120	1; 0.83 (r)	
Kaou	115	14; 12.17 (d)	2.381, 0.017
	61	1; 1.64 (r)	

*Abbreviations* d, dry season; r, rainy season

### Association of trypanosome infection with anaemia

A total of 39 cattle were anaemic against 867 non-anaemic. The prevalence of trypanosome- infected cattle was 41.02 % and 7.62 % in anaemic and non-anaemic cattle, respectively. Anaemia was positively correlated with trypanosome infection (OR: 5.259; 95 % CI: 3.356–8.216). This association was observed in both anaemic and non-anaemic cattle during the dry and the rainy seasons (Table 2). The sensitivity of the PCV test was 20 % at 24 % value of PCV. The sensitivity increased with the PCV: 26 % (16/78), 54 % (42/78) and 77 % (60/78) at 26 %, 28 % and 30 % PCV, respectively.

**Table 2:** Effect of season on anaemia status and trypanosome infection

Season	Animal blood status	Infected	Non infected	Total	OR(95 % CI)
Dry season	Anaemic (PCV ≤ 24)	14	15	29	4.564 (2.904–7.170)
	Non anaemic (PCV > 24)	54	465	520	
Rainy season	Anaemic (PCV ≤ 24)	2	8	10	7.675; 1.863–31.617
	Non anaemic (PCV > 24)	8	299	307	

*Abbreviations*: OD, Odds ratio; CI, Confidence interval

### Species of trypanosomes

Three species of trypanosomes were identified: *Trypanosoma vivax, T. brucei* and *T. congolense. Trypanosoma vivax* was the most abundant species with 73.23 % (52/71) (*χ*^2^ = 76.605, *P* = 0.0001).The prevalence of *T. congolense* was 15.49 % (11/71) while that for *T. brucei* was 11.27 % (8/71).

### Effect of breed and age on trypanosome infection

The highest likelihood for a clinically healthy animal to be free from trypanosome infection was recorded in the Gudali breed, followed by the Charolais × gudali, the white Fulani and the red Fulani in that order (Table 3).

**Table 3:** Effect of cattle breed on trypanosome infection rate

Breed	Infected (%)	Non-infected	Total	OR (95 % CI)
Gudali	46 (6.48 %)	663	709	14.41, 10.69–19.43
Charolais-gudali	17 (15.38 %)	76	93	5.5, 1.22–24.81
White Fulani	13 (18.27 %)	38	51	4.47, 2.64–7.56
Red Fulani	2 (25.49 %)	11	13	2.92, 1.55–5.49

*Abbreviations*: OD, Odds ratio; CI, Confidence interval

### Effect of trypanosome infection on PCV and BCS

Cattle infected with trypanosomes had a mean PCV of 28.95 (SD = 4.34) whereas that for cattle not infected with trypanosomes was 34.05 (SD = 5.70).This difference in PCV was statistically significant (*t*_(44) =_ -7.696, *df =*1, *P =* 0.001). The BCS was significantly (t: -4.350; *df = 2, p* < 0.001) lower in cattle infected with trypanosomes (Mean: 3.28; SD = 0.44) than in trypanosome free cattle (Mean: 3.53; SD = 0.48). In the dry season trypanosome-infected cattle had a significantly (*P* < 0.001) lower BCS than uninfected cattle whilst in the rainy season there was a non-significant (*P* > 0.05) difference between trypanosome infected cattle and trypanosome-free cattle for BCS. In the dry season trypanosome-infected cattle had a significantly (*P* < 0.001) lower PCV than trypanosome-free cattle whilst in the rainy season the difference in PCV between trypanosome-infected cattle and trypanosome-free cattle was not significant (*P* > 0.05) (Table 4).

**Table 4:** Trypanosome infection with respect to BCS, PCV and season

	Season	Mean (SD)	*t*-value, *P*-value
BCS	Dry season	3.25 (0.45)^a^	-3.266, 0.001
		3.44 (0.46)^b^	
	Rainy season	3.50 (0.39)^a^	-1.161, 0.247
		3.67 (0.48)^b^	
PCV	Dry season	28.52 (3.51)^a^	-7.893, 0.001
		34.67 (6.24)^b^	
	Rainy season	31.55 (7.435)^a^	-1.071, 0.285
		33.08 (4.566)^b^	

^a^Trypanosome-infected cattle; ^b^Trypanosome-free cattle

### Entomological results

**Fig. 2:**
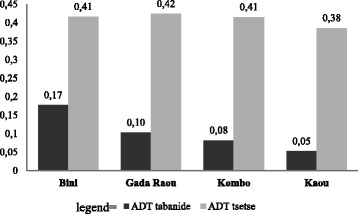
Tsetse and tabanids Apparent Density of Trap (ADT)per sampling sites

Four hundred and ninety three haematophagous flies were trapped: 390 tsetse flies and 103 Tabanids. The mean ADT was 1.09 (SD = 0.91) with biconical traps and 2.16 (SD = 1.34) with Nzi traps. The Nzi trap caught significantly more haematophagous flies than the biconical traps (*t*_(44)_ = -3.664, *df* =2, *P* = 0.001). Abundance of flies was not significantly different among the sampled villages for both tabanids (*F* = 2.429, *df* =16/12, *P* = 0.094) and tsetse (*F* = 0.60, df = 1000/200, *P* = 0.980) (Fig. 2). Haematophagous flies were significantly (*t*_(44)_ = −2.216, df = 4, *P* = 0.03) more abundant at altitudes lower than 730 m (1.655 individuals; SD = 1.325) than at higher altitudes (1.09 individuals; SD = 0.866). Two species of tsetse flies were collected and identified in the sampling sites: *Glossina tachinoides* and *G.morsitans submorsitans*; the latter was the more prevalent species with 66. 41 % (259/390) against 33.59 % (131/390) for *G. tachinoides* (*Z* = 9.166; *P* = 0). Female flies represented 71.79 % (280/390) of the tsetse population against 28.21 % for males (110/390) (*Z* = 12.174; *P* = 0)

### Prevalence of trypanosome infection in tsetse flies

Out of the 390 tsetse flies trapped, 194 non-teneral flies were dissected: 122 *G. morsitans* (81 female and 41 male) and 72 G*. tachinoides* (53 female and 19 male); nine of them (4.64 %) were infected with trypanosomes. The infection rate appeared slightly higher among individuals of *G. morsitans* (5.74 %; 7/122) than those of *G. tachinoides* (2.78 %; 2/72) but this difference was not significant (Z = 0.946, *P* = 0.342). There were as many female (3.73; 5/134) as male (3.33 %; 2/60) infected (*Z* = 0.137, *P* = 0.888). Three species of trypanosomes were found: *T. vivax* (66.67 %; 6/9), *T. brucei* (22.22 %; 2/9) and *T. congolense* (11.11 %; 1/9); *T. vivax* was the most prevalent species. Infected tsetse flies were found in three of the survey zones: Bini (7.57 %; 5/66), Gada Raou (2.77 %; 2/72) and Kombo (5. 71 %; 2/35); these differences in prevalence were not statistically significant (*X*^2^ = 1.630, *df* = 6, *P* = 0.442).

## Discussion

The cattle examined for animal trypanosomosis in the present study were exposed to trypanosome transmission risk for three months immediately after receiving prophylactic doses of a trypanocide (Isometamidium) and had no clinical signs of trypanosomosis. In this background an epidemiological study involving the parasite’s definitive host and its vectors was undertaken in a ranch (Cambeef) in north Cameroon. Less than 5 % of animals examined had PCV values not higher than 24 %. Two conclusions could be drawn from this result. The first point is the robustness and reliability of the eye color chart for the detection of anaemia in cattle as reported by Grace et al. [22]; it stands as an alternative to PCV. The second point is herdsmen’s good ethno-veterinary knowledge [23, 24]. The noticeable difference of BCS and PCV values observed among cattle between seasons may be a consequence of the variations in the amount of fodder available in the different seasons. There is more fodder in the rainy season than in the dry season [25, 26]. The presence of trypanosome-infected animals among examined cattle may indicate a prophylactic drug failure and implies the existence of a resistant strain of trypanosomes circulating in the study zone. Resistance to Isometamidium was already reported by Mamoudou et al. [27] in north Cameroon. The fact that drugs are administered by trained veterinarians in the ranch suggests that the prophylactic failure may lie either on the repeated use of the same molecules of trypanocide [28, 29] and/or on the quality of drug used. Vougat et al. [30] reported that many veterinary medicines in the market and veterinary pharmacies are not up to the standards required in north Cameroon. This fact presumes that the risk for the development of drug resistance is likely higher among traditionally managed livestock where the majority of pastoralists administer drugs without any parasitological diagnosis or even clinical examination [31] and without respect of drug prescription [30, 32, 33]. There is a need to evaluate the current levels of trypanosome drug resistance in the region and its impact on cattle management. The other point that could be drawn from this survey is the existence of an important reservoir of trypanosomes left within herds of the ranch particularly at the end of the dry season which contributes, certainly, to the endemicity of the disease in the studied zone where game animals are rare.

The overall prevalence of bovine trypanosomosis (9 %) in the study area is lower than that reported by Mamoudou et al. [3] in the same zone. The lower trypanosomosis prevalence in this study is simply the result of the use of different sampling procedures. In the previous study there was no discrimination of animals according to their clinical status. However, the present prevalence rate is higher than that observed by Dinsa et al. [34] (2.20 %) and Mulaw et al. [35] (1.8 %) among cattle of good BCS in Ethiopia. This may be due to the difference in the virulence of trypanosome strains. The prevalence of bovine trypanosomosis was more than three times higher during the dry season than the rainy season. This difference could be explained by seasonal migration, reported as a risk factor by Delafosse et al. [36]. Animals of the ranch migrate far into the ranch in search of fodder gathering around the few water points left where contact with haematophagous flies is high.

In our study, the impact of trypanosome infection as an animal health indicator was found to be significant, notably in the dry season. The non-significance of this impact in the rainy season is due to the increased resilience of animals as a result of the better nutrition [37] during the rainy season. The likelihood for an apparently healthy animal to be parasitologically free of trypanosome infection is at least three times higher in the Gudali breed when compared with the white and red Fulani; this difference could denote an increased tolerance of the Gudali to the strain of trypanosome in circulation in the zone. Anaemia is found to be positively correlated with trypanosome infection. Other studies have already reported this association [38, 39]. Anaemia is a well-recognized and inevitable consequence of an infection with pathogenic trypanosomes [40]; it is measured by PCV which stands as a reliable tool for detecting trypanosome-infected animals in the absence of other factors causing anaemia [22, 41]. The PCV sensitivity for values of 24 % and 26 % was very low as compared to that obtained by Marcotty et al. [42], but sensitivity increases to 54 % and even 77 % using a PCV cut-off of 28 % or 30 %, respectively. The level of anaemia at which an animal could be considered infected with trypanosomes is higher for asymptomatic cattle and it is crucial, for the reliability of this test, to always establish the cut-off value prior to its use for diagnosis [42]. Diagnosis and treatment of asymptomatic animals among apparently healthy cattle alongside the symptomatic animals of the ranch is the implicit response for considerably reducing the reservoir of trypanosomes within the herds and controlling the disease. A mass-screening of the herds once every year with a follow-up of drug sensitivity testing could reinforce the control approach in this setting. In the present study, infections were largely dominated by *T. vivax.* Similar observations were made by Ndamkou and Chare [2] in the North region and by Mpouam et al. [43] in the Vina division, Adamawa region, Cameroon. This dominance of *T. vivax* suggests an important contact of cattle with tsetse flies, efficient vector of this species [44] and/or tabanids [45]. Besides, *T. vivax* is known to have a 25-fold higher chance for mechanical transmission than *T. congolense* [46].The entomological survey revealed the presence of Glossininae and Tabaninae flies in the ranch. Tsetse flies dominated with nearly 80 % of the total fly catch. The apparent densities of both flies were similar in the different villages. The Nzi traps were significantly more effective than biconical traps. The same observation was made in another study [47]. The larger surface of the Nzi trap as compared to the biconical trap could be the determinant factor as reported by Phelps, [48] and Ryan, [49] who observed that efficacy increases with the size of the trap.

*Glossina morsitans* and *G. tachinoides* were the two species of tsetse fly identified in the study area; these species are adapted to the savannah zone [50] and are known to be abundant in the north of Cameroon [51]; *G. tachinoides* is, contrary to *G. morsitans submorsitans*, was confined to forest galleries. The difference in abundance observed between these two tsetse fly species may be due to the habitat factor and/or altitude; the lowest altitude recorded where traps were set is 607 m and according to Rageau & Adam [51] and *G. tachinoides* are hardly found above 600 m of altitude. Regardless of the species of haematophagous flies, the abundance of flies was significantly higher at altitudes lower than 730 m. Climate, which largely depends on altitude, influences fly abundance as reported by Leak [52].

The fly sex ratio was assessed and there were more female than male flies. Similar results were obtained by Morlais [53] and Tchouomene-Labou et al. [54]. This situation seems to be the rule. According to Leak’s [52] observations, female would comprise between 70 to 80 % of the mean population in an unbiased sample. Trypanosome prevalence was not significantly different between female and male flies and the slight increase of infection in female may be due to the higher lifetime and aggressiveness of female as compared to male flies [53, 55]. Both species of tsetse fly were found infected with trypanosomes although the infection rate was significantly higher in *G.morsitans submorsitans* than in *G. tachnoides.* Desta et al. [56] made similar observations in western Ethiopia. This difference could be due to their habitat.

## Conclusions

In conclusion, carriers of trypanosomes are effectively present among apparently healthy cattle. Their existence, a few months after prophylactic treatment against trypanosomosis, suggests that a resistant strain of trypanosomes may be circulating within the ranch and thus sustains the endemicity of the disease.
